# Correction: Chemical profile and analysis of biosynthetic pathways and genes of volatile terpenes in *Pityopsis ruthii*, a rare and endangered flowering plant

**DOI:** 10.1371/journal.pone.0333001

**Published:** 2025-09-19

**Authors:** Xinlu Chen, Marcin Nowicki, Phillip A. Wadl, Chi Zhang, Tobias G. Köllner, Miriam Payá‐Milans, Matthew L. Huff, Margaret E. Staton, Feng Chen, Robert N. Trigiano

In Fig 1, the parts of the panel B are duplicated. Please see the correct Fig 1 here.

**Fig 1:**
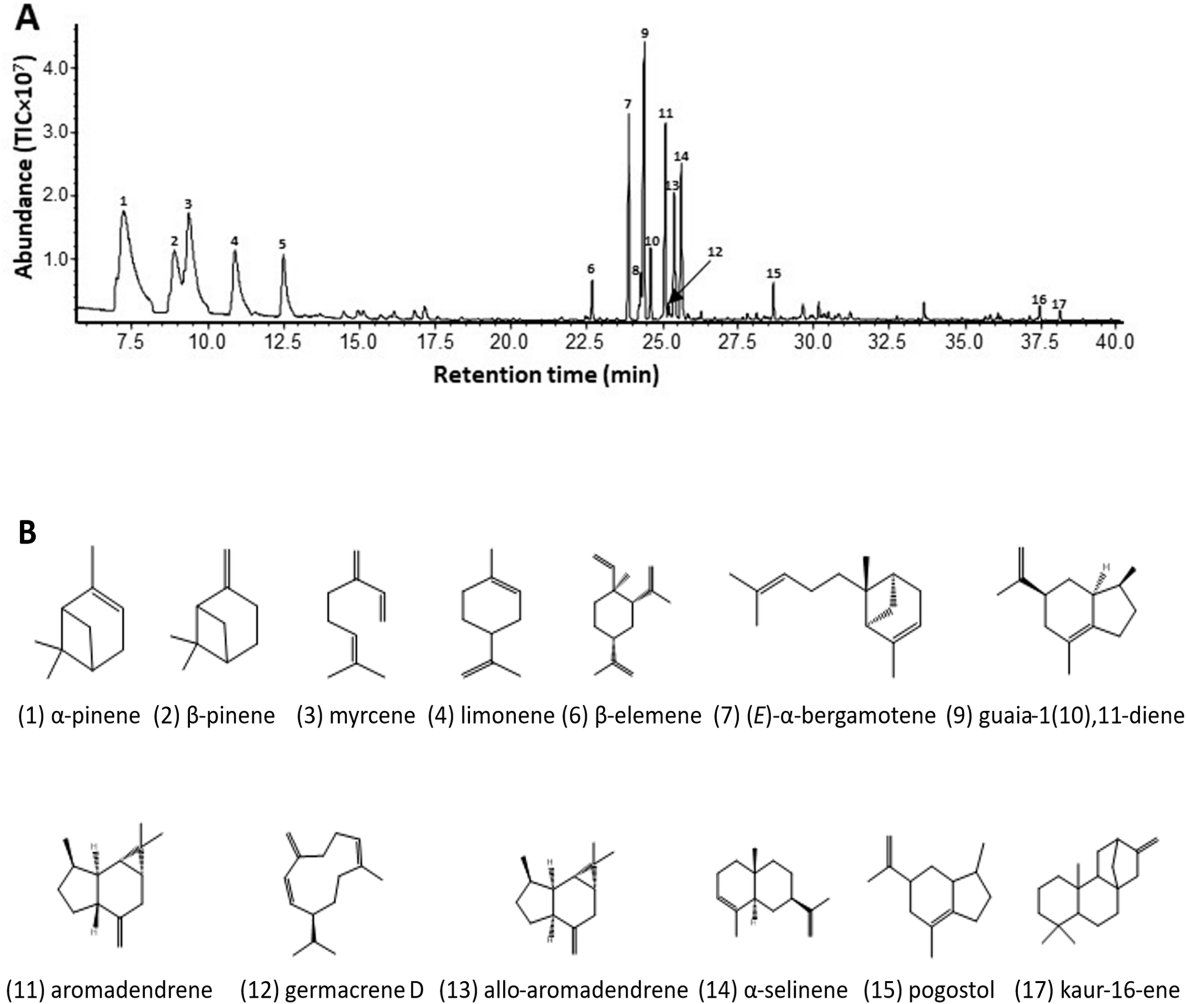
Gas chromatograms of terpene volatiles emitted from open flowers and leaves of Pityopsis ruthii.

There are a number of errors in the caption for Fig 2, “Terpene pathways involved in terpene biosynthesis in Pityopsis ruthii.” Please see the complete, correct Fig 2 caption here.

**Fig 2:**
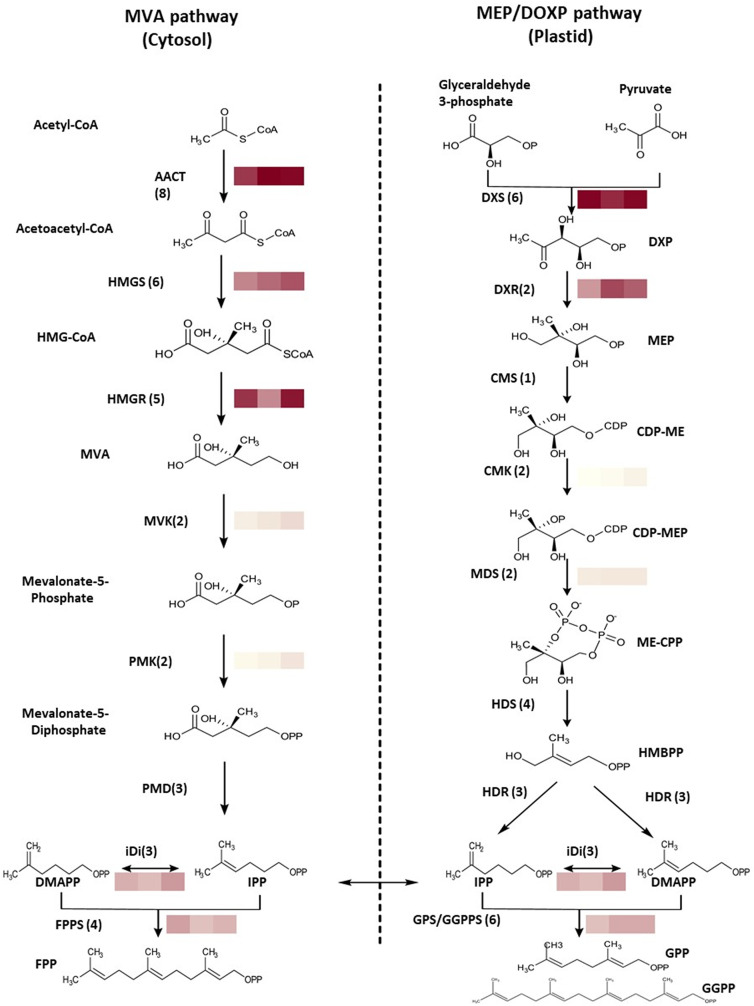
Terpene pathways involved in terpene biosynthesis in Pityopsis ruthii. Heatmap of the data from flower transcriptome analyses performed in triplicate along with representative gene indicated expression level, which was calculated with fragments per kilobase of transcripts per million mapped fragments (FPKM). Abbreviations of genes: AACT, acetoacetyl-CoA thiolase; HMGS, hydroxylmethylglutaryl-CoA synthase; HMGR, hydroxymethylglutaryl-CoA reductase; MVK, mevalonate kinase; PMK, 5-phospho-mevalonate kinase, PMD, mevalonate diphosphate decarboxylase; FPPS, farnesyl pyrophosphate synthase; DXS, 1-deoxy-d-xylulose-5-phosphate synthase; DXR, 1-deoxy-d-xylulose-5-phosphate reductoisomerase; CMS, 2-C-methyl-d-erythritol 4-phosphate cytidylyltransferase; CMK, 4-diphosphocytidyl-2-C-methyl-d-erythritol kinase; MDS, 2-C-methyl-D-erythritol 2,4-cyclodiphosphate synthase; HDS, **(E)**-4-hydroxy-3-methylbut-2- enyl diphosphate synthase; HDR, 4-hydroxy-3-methylbut-2-enyl diphosphate reductase; IDI, isopentenyl-diphosphate delta-isomerase; GPPS, geranyl diphosphate synthase. Compound abbreviations: HMG-CoA, 3-hydroxy-3-methylglutaryl-CoA; MVA, mevalonate; DXP, 1-Deoxy-D-xylulose 5-phosphate; MEP, 2-C-Methyl-D-erythritol 4-phosphate; CDP-ME, 2-C-Methyl-d-erythritol-2,4-cyclodiphosphate; CDP-MEP, 2-Phospho-4-(cytidine 5’-diphospho)-2-C-methyl-D-erythritol; ME-CPP, 2-C-methyl-d-erythritol-2,4-cyclodiphosphate; HMBPP, 1-hydroxy-2-methyl-2-(E)-butenyl-4-diphosphate; FPP, **(E,E)**-farnesyl pyrophosphate; IPP, isopentenyl pyrophosphate; DMAPP, dimethyallyl pyrophosphate; GPP, geranyl pyrophosphate; GGPP, geranylgeranyl pyrophosphate. Color scale used shows transcript abundance and ranges from 307 (CMK_1) to 6727 (AACT_2) FPKM.
