# Supplementary figures and images for: Chemical profile and analysis of biosynthetic pathways and genes of volatile terpenes in Pityopsis ruthii, a rare and endangered flowering plant
Source: PLoS One. 2023 Jun 23;18(6):e0287524. doi: 10.1371/journal.pone.0287524 (PMC10289357; doi:10.1371/journal.pone.0287524)

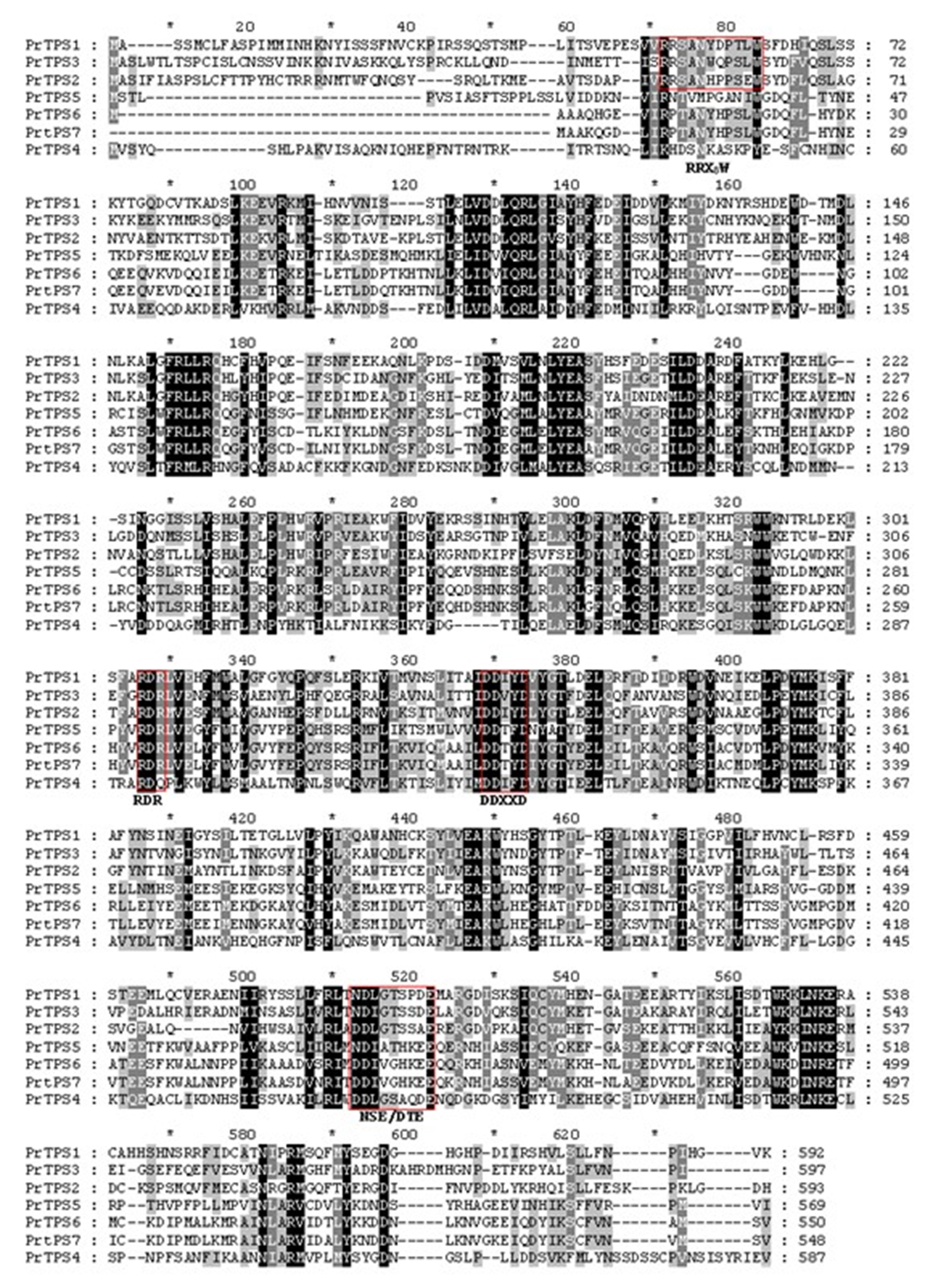

Supplement: S1 Fig — Identical amino acids are highlighted with increasingly darker backgrounds. Motifs typically found in TPSs are boxed with red boarders, and the respective motif names are identified below. (TIF) [file pone.0287524.s004.tif]

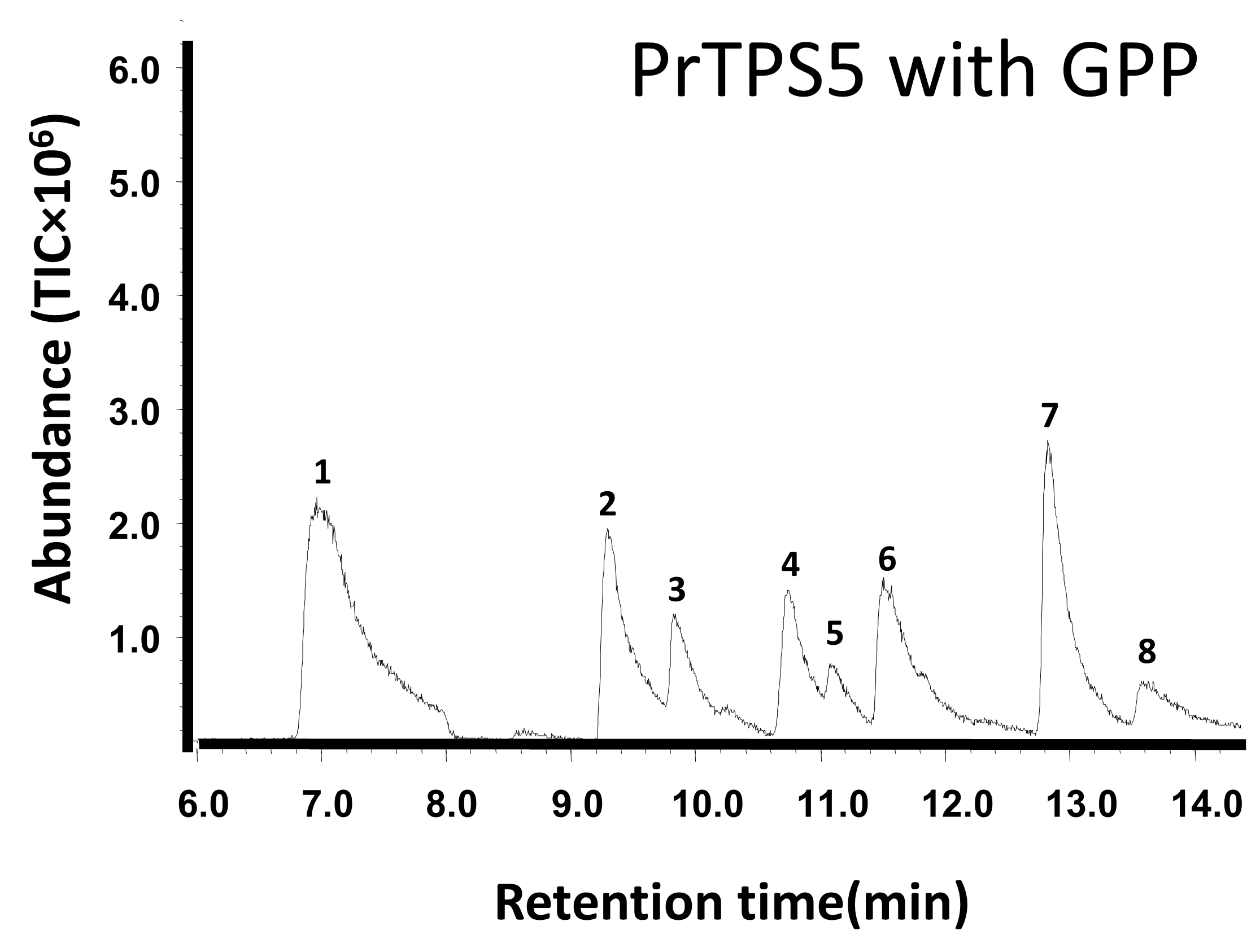

Supplement: S2 Fig — Crude proteins extracted from heterologous expression in E. coli catalyzed the conversion of the substrate GPP into monoterpenes. Products identified by GC-MS: 1. α-pinene; 2. Myrcene; 3. α-phellandrene; 4. Limonene; 5. (Z)-β-ocimene; 6. (E)-β-ocimene; 7. α-terpinolene; 8. unidentified monoterpene. (TIF) [file pone.0287524.s005.tif]
