# Supplementary material for: Chemical profile and analysis of biosynthetic pathways and genes of volatile terpenes in Pityopsis ruthii, a rare and endangered flowering plant
Source: PLoS One. 2023 Jun 23;18(6):e0287524. doi: 10.1371/journal.pone.0287524 (PMC10289357; doi:10.1371/journal.pone.0287524)
Supplement: Attachment — Submitted filename: Reviewers Comments.docx [file pone.0287524.s006.docx]

**Comments to the Author**

Chemical profile and biosynthesis of volatile terpenes in Pityopsis ruthii, a rare and endangered flowering plant by Chen et.al., reported important information about a rare and endangered plant to improve its conservation efforts. This report depicts some new information regarding the biosynthesis of floral terpenes in Pityopsis ruthii which can be beneficial for chemical defense mechanisms and the attraction of pollinators. Based on the results, I speculate that the authors analyzed the terpene profiles from floral parts of the plant and studied genes involved in terpene biosynthesis. They further confirmed the involvement of these genes by studying the metabolic pathway of terpene biosynthesis.  This information can be helpful in the assessment of how the terpenes may help the plant survive in different habitats.

Although this study is very important for the conservation of this plant, but only studying terpene biosynthesis is not enough to decide about the survival of the plant in different habitats. As the authors mentioned in the introduction part, this plant reached to end line because it is very tough for this plant to survive in the presence of other species. There is also no explanation that why only this specific part of the United States is promoting the growth of this plant and if we try to grow this plant in any other area, these terpene biosynthesis genes would be enough to do this. What is the purpose of studying only the terpene biosynthesis gene of already endangered plants if the plant’s growth and survivability are questioned at the end???

**Title:** The title is quite misleading according to the findings of the paper, as there is no biosynthesis of terpenes, instead analysis of biosynthetic pathways and genes

**Abstract:** The objective of the paper is not mentioned in the abstract which is an important part of the abstract. The authors only mentioned the results and conclusion.

**Introduction**: The introduction is good but needs to incorporate a few lines regarding the adaptation of this plant in other habitats and what else can be beneficial for their adaptation. In the introduction, the only focus is on terpene biosynthesis but there is a need to incorporate some other basic adaptation requirements. Please try to use the third form in writing papers as it is mentioned in the last paragraph of the introduction.

**Materials and Methods:** This section is written in detail and all the information has been provided to repeat the experiment. However no statistical analysis performed in the paper. Statistical data should provide for table 1.

**Results:** Well written. In every section, the authors mentioned references to support their data. As this journal has a separate discussion part, it will be good to justify your findings in the discussion section and only mention your results in the result section to avoid confusion. Quality of Figures 1 and 2 needs improvement as it is very hard to read small letters. Explain in one or two lines about the function and origin of all studied genes to make it easy to understand for the reader.

**Discussion:** Well written and results are well justified. Please try to use the third form in writing papers as there are multiple sentences in the discussion where authors used the first form.

**Conclusion:** Conclusion is missing in this paper. Authors should add a conclusion section to highlight the findings of the paper in 1 or 2 paragraphs.
